# A Kelch domain-containing KLHDC7B and a long non-coding RNA ST8SIA6-AS1 act oppositely on breast cancer cell proliferation via the interferon signaling pathway

**DOI:** 10.1038/s41598-018-31306-8

**Published:** 2018-08-27

**Authors:** Gookjoo Jeong, Hansol Bae, Dawoon Jeong, Juyeon Ham, Sungbin Park, Hyeon Woo Kim, Han-Sung Kang, Sun Jung Kim

**Affiliations:** 1 Department of Life Science, Dongguk University-Seoul, Goyang, Republic of Korea; 2 Research Institute and Hospital, National Cancer Center, Goyang, Republic of Korea; 3 Present Address: PanGen Biotech Inc, Suwon, 16675 Republic of Korea

## Abstract

In our previous study, the Kelch domain-containing 7B (KLHDC7B) was revealed to be hypermethylated at the promoter but upregulated in breast cancer. In this study, we identified a long non-coding RNA, ST8SIA6-AS1 (STAR1), whose expression was significantly associated with KLHDC7B in breast cancer (*R*^2^ = 0.3466, *P* < 0.01). Involvement of the two genes in tumorigenesis was examined via monitoring their effect on cellular as well as molecular events after each gene dysregulation in cultured mammary cell lines. Apoptosis of MCF-7 decreased by 49.5% and increased by 33.1%, while proliferation noted increase and decrease by up- and downregulation of KLHDC7B, respectively, suggesting its oncogenic property. STAR1, however, suppressed cell migration and increased apoptosis. Network analysis identified many target genes that appeared to have similar regulation, especially in relation to the interferon signaling pathway. Concordantly, expression of genes such as IFITs, STATs, and IL-29 in that pathway was affected by KLHDC7B and STAR1. Taken together, KLHDC7B and STAR1 are both overexpressed in breast cancer and significantly associated with gene modulation activity in the interferon signaling pathway during breast tumorigenesis.

## Introduction

During development of breast cancer, as in other cancer types, tumor suppressors or oncogenes are deregulated, resulting in abnormal cell proliferation^1^. Many genes have been identified to be responsible for cell division, proliferation, or apoptosis during tumorigenesis^2^. Oncogenes and tumor suppressors can be either activated or inactivated by mutations such as gene rearrangement, deletion, insertion, or substitution. In addition to genetic causes, epigenetic regulation of tumor-related genes has been discovered, including methylation of CpG sites, regulation by microRNA or long non-coding RNA (lncRNA), and histone modification^3^. For methylation of CpG, hypermethylation generally inactivates tumor suppressors such as p53^4^ and BRCA1^5^, while hypomethylation activates oncogenes such as MET^6^ and SNCG^7^ in breast cancer. The alteration of methylation levels can occur on miRs and lncRNAs as well as protein-coding genes. In breast cancer, miR-375^8^ and linc00152^9^ undergo hypomethylation at the promoter and thereafter stimulate cell proliferation. Both miR-192-2^10^ and ZNF667-AS1^11^ are known as tumor suppressors; they are inactivated in breast cancer through hypermethylation at their promoter.

KLHDC7B was originally identified as a 3008-bp long expressed sequence tag (EST). The transcript is translated into a 595 amino acids-long protein (https://www.proteinatlas.org) containing a Kelch domain that is comprised of a four-stranded β-sheet of the Kelch motif to form one blade of a β-propeller structure^12^. Kelch-containing proteins have roles in extracellular communication/interaction, cell morphology, gene expression, and actin binding; they can be co-opted by a virus after an infection. A well-known Kelch-containing protein is KEAP, which negatively controls the NRF2 transcription activator^13^. Some of the genes controlled by NRF2 are important for cellular defense against harmful oxidative stresses, possibly resulting in carcinogenesis; this implies a KEAP1 loss of function can facilitate cancer cell expansion. In contrast to other proteins in that class, the detailed protein structure and function of KLHDC7B in normal cells, as well as cancer cells, has yet to be determined. In our previous study, KLHDC7B was identified as a potential epigenetic marker, showing hypermethylation in the promoter in breast cancer cell lines as well as in cancer tissues^14^. The gene is one of the rare hypermethylated examples, which is nevertheless upregulated in cancer tissue.

The lncRNAs are ~200 bp RNA compounds that are not encoded into protein. In breast cancer research, many lncRNAs have been revealed to play pivotal roles in stimulating or suppressing cancer cells, acting alone or in combination with proteins^15^. They are involved in cell growth, apoptosis, cell migration, and invasiveness, as well as cancer cell development. H19 is one of the most well-characterized lncRNAs; it is encoded by the maternal allele and capable of silencing the IGF2 gene in the same allele^16^. H19 is abnormally overexpressed in cells of higher tumorigenic capacity^17^ and therefore considered as an oncogenic RNA. In contrast, GAS5 causes growth arrest and induces apoptosis in breast cell lines, and it is significantly downregulated in breast cancer cells^18^.

In this study, genes affected by KLHDC7B were identified in the MCF-7 breast cancer cell line after inducing up- and downregulation of the gene. Among the affected, a lncRNA, ST8SIA6-AS1 (STAR1 hereafter), showed a concomitant change in expression by KLHDC7B. Both KLHDC7B and STAR1 were upregulated in breast cancer tissue. This study examined the effects of KLHDC7B and STAR1 on cellular activities including apoptosis, proliferation, and migration. By analyzing the target genes commonly regulated by KLHDC7B and STAR1, interferon signaling was identified as the key pathway. Our results suggest a significant association of expression between KLHDC7B and STAR1 in breast cancer. This study discussed the contribution of the two genes to the fine control of cancer cell proliferation.

## Results

### Expression of KLHDC7B and STAR1 is significantly associated in breast cancer

**Figure 1:**
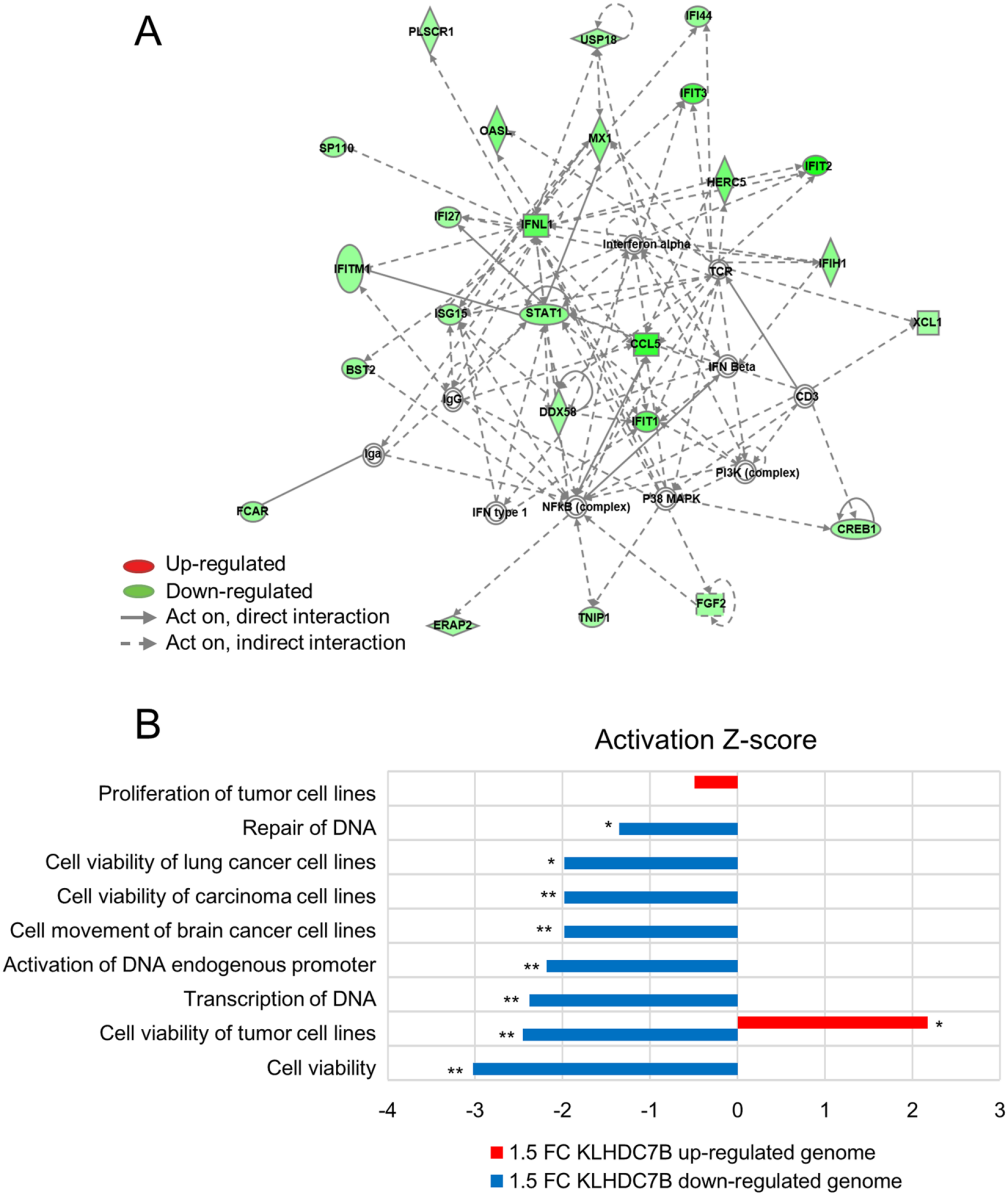
Functional annotation of genes displaying altered expression by dysregulation of KLHDC7B in MCF-7. (**A**) The highest confidence network of genes displaying altered expression by downregulation of KLHDC7B in MCF-7. KLHDC7B was downregulated in MCF-7 by transiently transfecting a siRNA and microarray analysis. The 188 genes that were deregulated with an expression change higher than 1.5-fold were analyzed by IPA. The highest confidence network is relevant to “Antimicrobial Response, Inflammatory Response, and Infectious Disease”. Genes that were upregulated are shaded in red, while downregulated genes are shaded in green, with an intensity signifying the magnitude of expression change. Solid and dashed lines represent direct and indirect interactions, respectively. (**B**) Top annotated disease and function obtained by IPA analysis. Disease and functional effects were analyzed with IPA for the genes that have shown deregulation by downregulation (blue bar) and upregulation (red bar) of KLHDC7B in MCF-7. The activation Z-score indicates the predicted state of related potential diseases or function to the identified genes.

**Figure 2:**
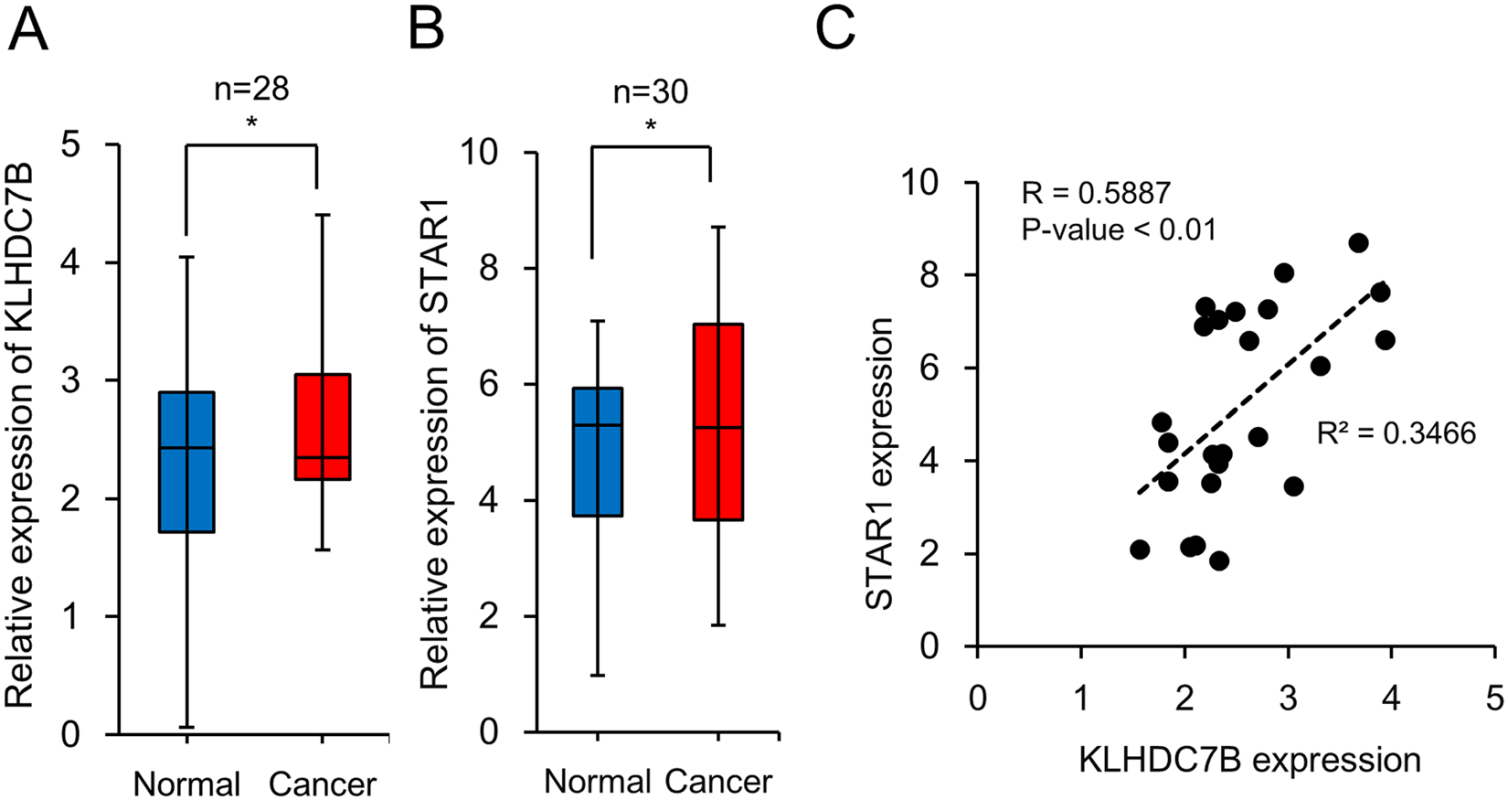
Expression of KLHDC7B and STAR1 is significantly associated in breast cancer. Expression of KLHDC7B (**A**) and STAR1 (**B**) was examined by real-time RT-PCR in breast cancer tissue and its nearby normal tissue. The result is represented by 2^−ΔCt^ method and shown by box plots. Both genes were revealed to be upregulated in the cancer tissue with a statistical significance (*P* < 0.05) when tested by Mann-Whitney U-test. (**C**) The expression association between KLHDC7B and STAR1 in cancer tissue was examined using the coefficient of determination (*R*^2^) calculated by linear regression (*R*^2^ = 0.3466, *P* < 0.01, N = 24).

KLHDC7B was identified in our previous study to be hypermethylated at the promoter and upregulated in breast cancer patients^14^. In the current study, to address its role during carcinogenesis, the gene was ectopically deregulated in mammary cell lines for examination of molecular and cellular changes. First, the gene was downregulated in the MCF-7 using a siRNA, which is a breast cancer cell line and expresses KLHDC7B at an elevated level (Supplemental Fig. S1A). Genome-wide gene expression assay was performed on a microarray that covered more than 47,300 human targets. 188 genes showing a statistically significant expression difference >1.5 fold were selected. Pathway analysis showed the “Antimicrobial Response, Inflammatory Response, and Infectious Disease” as the top network (Fig. 1A). Next, KLHDC7B was overexpressed using an ORF-containing plasmid vector in MCF-7 (Supplementary Fig. S1A). Interestingly, a smaller number of affected genes was observed. Only 48 genes significantly changed relative to the overexpression of KLHDC7B compared to the downregulation. The small number of gene set was not enough to draw an IPA pathway, however, the genes mainly comprised of those tending to increase tumor cell viability (Fig. 1B). The genes deregulated by downregulation of KLHDC7B changed toward decreasing “cell viability of tumor cell lines” and “repair of DNA”; meanwhile, the genes deregulated by upregulation of KLHDC7B changed toward increasing “cell viability of tumor cell lines” as judged by the IPA disease and function analysis (Fig. 1B). Notably, among the dysregulated genes, a long non-coding RNA (lncRNA), ST8SIA6-AS1 (also named as STAR1 in this study, of which the function is yet to be known), commonly appeared with KLHDC7B overexpression and downregulation (Supplemental Fig. S2A). The lncRNA was increased 1.57 fold by overexpression but decreased 1.5 fold by downregulation of KLHDC7B, suggesting its possible regulation by KLHDC7B. Expression of STAR1 was upregulated in breast cancer cell lines (Supplementary Fig. S2B) and also in breast cancer tissue compared to its nearby normal tissue (N = 30, *P*-value < 0.05), similar to KLHDC7B (N = 28, *P*-value < 0.05) (Fig. 2A,B). An association of statistical significance was found between KLHDC7B and STAR1 expression in breast cancer tissues (R = 0.5887, *P*-value < 0.01) (Fig. 2C).

### KLHDC7B and STAR1 show opposite activity in cancer cell proliferation

**Figure 3:**
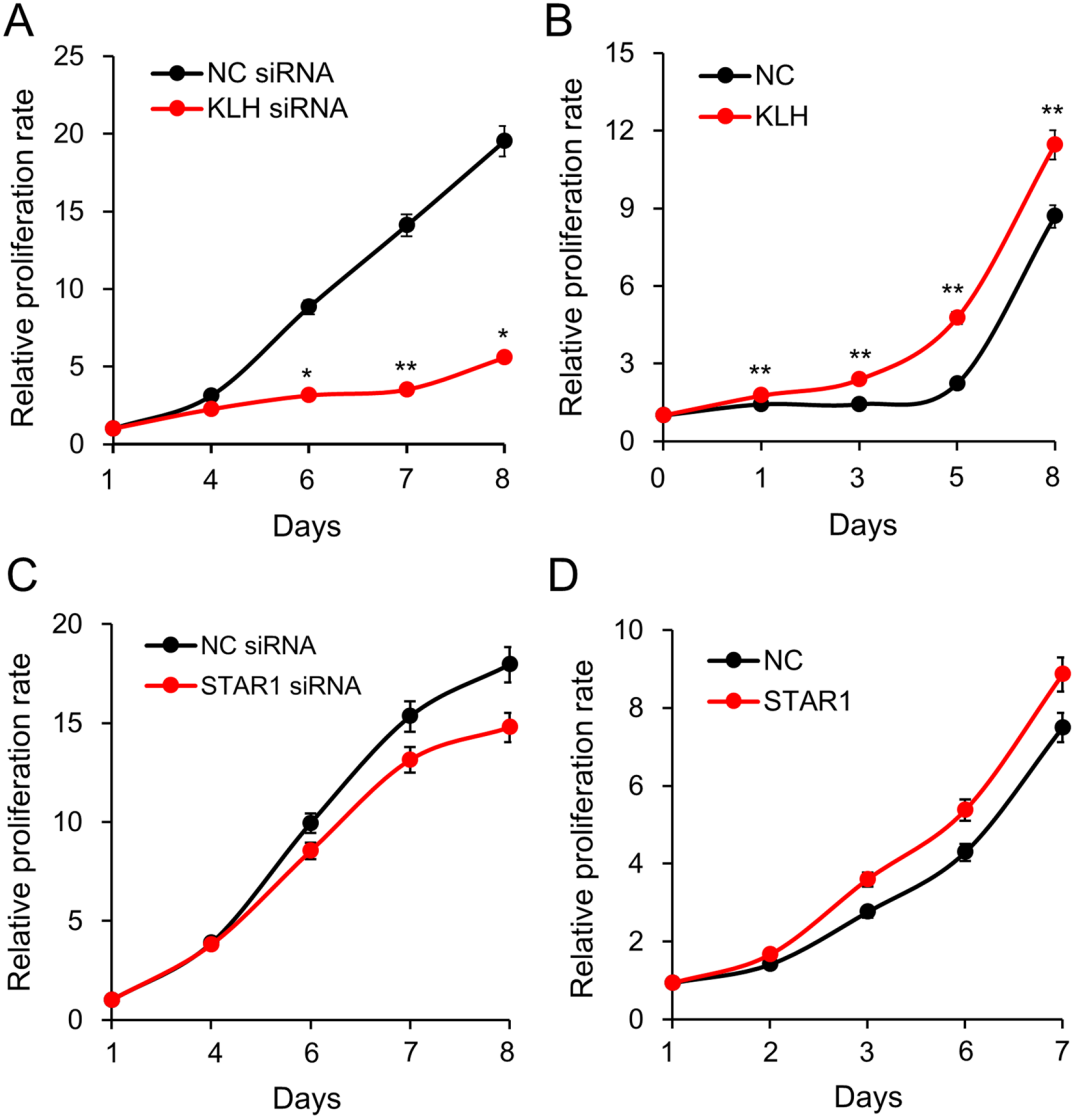
Effect of KLHDC7B and STAR1 on cell proliferation. The role of KLHDC7B and STAR1 in cell proliferation was examined by the dye-based CCK assay. KLHDC7B and STAR1 were induced to be downregulated (**A**,**C**) and upregulated (**B**,**D**) in MDA-MB-231 by transiently transfecting a recombinant plasmid vector and a siRNA. All the assays were performed in triplicate, and the result is depicted as Mean ± SE. NC, negative control vector or siRNA.

In order to examine the effect of KLHDC7B and STAR1 on the proliferation of cancer cells, the proliferation of MDA-MB-231 was analyzed after inducing each gene deregulation (Supplementary Fig. S1C,D). For KLHDC7B, downregulation by siRNA remarkably decreased cell proliferation by approximately 71% on day 8 compared to the control siRNA (Fig. 3A). Meanwhile, overexpression did not significantly affect proliferation, only inducing a slight increase (Fig. 3B). As STAR1 has a significant association of expression with KLHDC7B in breast cancer, a cell proliferation assay was carried out to compare their effect on cell growth. As results, proliferation and suppression of the cells were not significantly affected, showing only a slight change when STAR1 was downregulated and upregulated, respectively (Fig. 3C,D).

**Figure 4:**
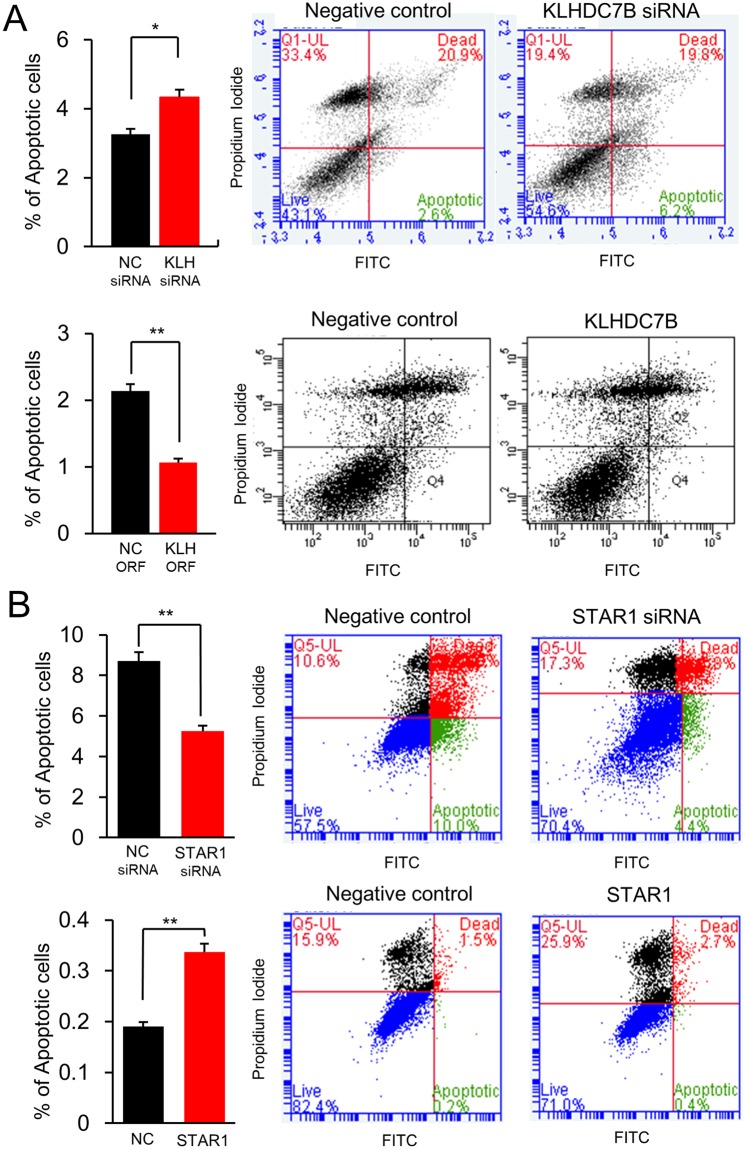
KLHDC7B increases apoptosis but STAR1 decreases apoptosis. Apoptosis of MDA-MB-231 cell was analyzed upon a flow cytometer after transiently transfecting a recombinant plasmid vector and a siRNA. Note (**A**) represents the apoptotic assay after downregulation and upregulation of KLHDC7B. Note (**B**) represents the apoptotic assay after downregulation and upregulation of STAR1. All the assays were performed in five independent experiments in triplicate, and the result is depicted as Mean ± SE. NC, negative control vector or siRNA.

In cell cycle analysis, KLHDC7B-upregulated cells did not show significant change of S phase (Supplementary Fig. S3A). On the other hand, the S phase was significantly increased in downregulation (Supplementary Fig. S3B). STAR1-upregulated cells showed an increase in the G0/G1 phase by 15.9% but STAR1-downregulated cells showed a decrease of G0/G1 by 10.6% (Supplementary Fig. S3C,D). Cellular apoptosis was increased in the KLHDC7B-downregulated cells by 33.1% but decreased in the KLHDC7B-upregulated cells by 49.5% (Fig. 4A). Cellular apoptosis was decreased 39.4% in the STAR1-downregulated cells but increased 78.9% in the STAR1-upregulated cells (Fig. 4B).

**Figure 5:**
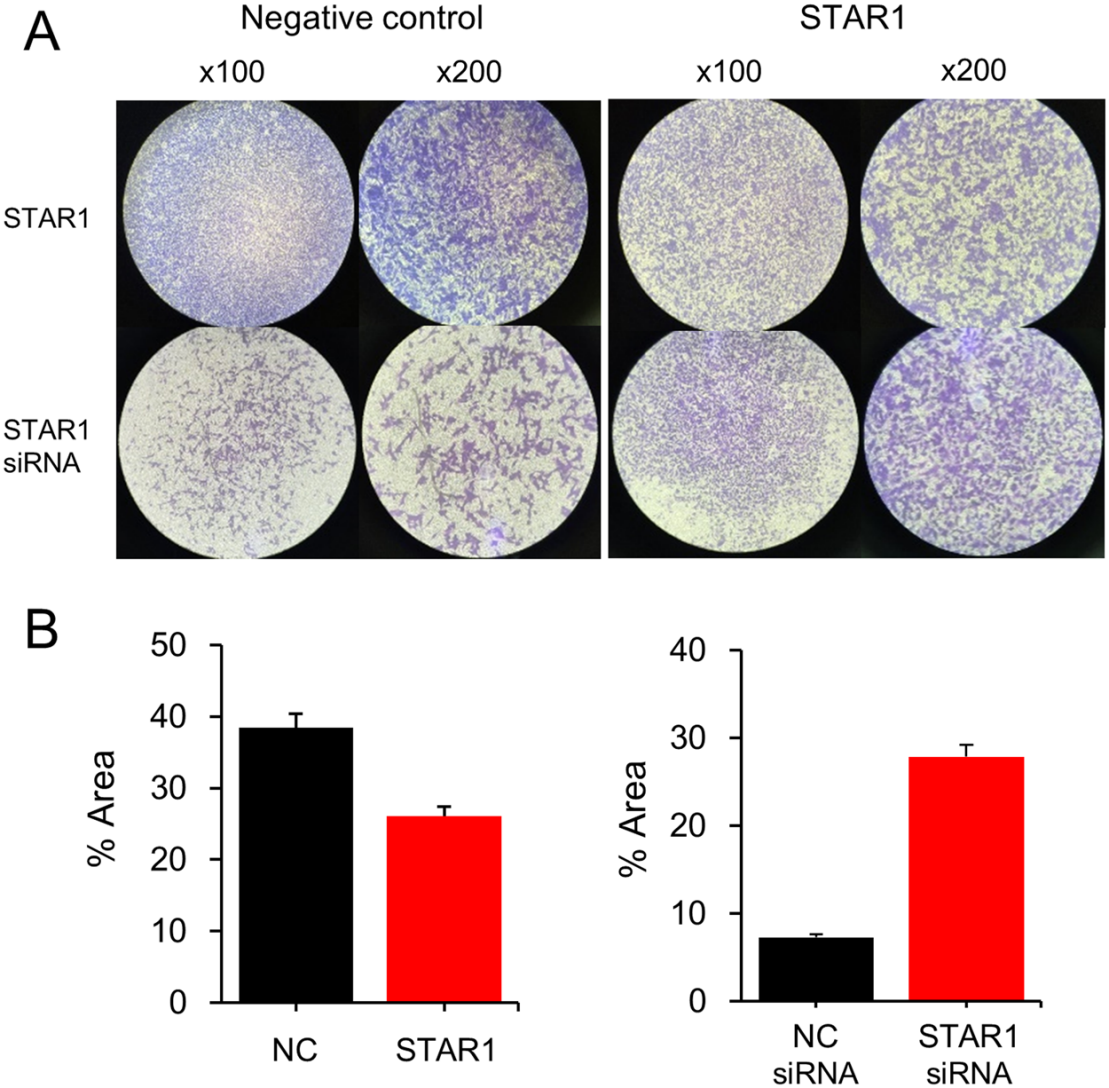
STAR1 decreases cell migration. Cell migration of MDA-MB-231 was examined after transiently transfecting a recombinant plasmid vector and a siRNA. Each experiment was carried out at least three times; representative images are shown (**A**). The migrated cells were analyzed using Image J software and shown as means with standard errors in a bar graph (**B**).

In order to see whether STAR1 participated in other cellular activities besides proliferation, a cell migration assay was performed for the STAR1-dysregulated MDA-MB-231 cell that was adopted instead of MCF-7, because the former has been known to be more invasive^19^. STAR1 overexpression decreased cell migration by 32.2% but its downregulation increased cell migration by 286% (Fig. 5). The result indicated that STAR1 is mainly involved in inhibiting cell migration rather than acting on cell proliferation. Taken together, KLHDC7B stimulated cell proliferation while STAR1 suppressed cell migration.

### Interferon signaling pathway is the key common regulatory pathway of KLHDC7B and STAR1

**Figure 6:**
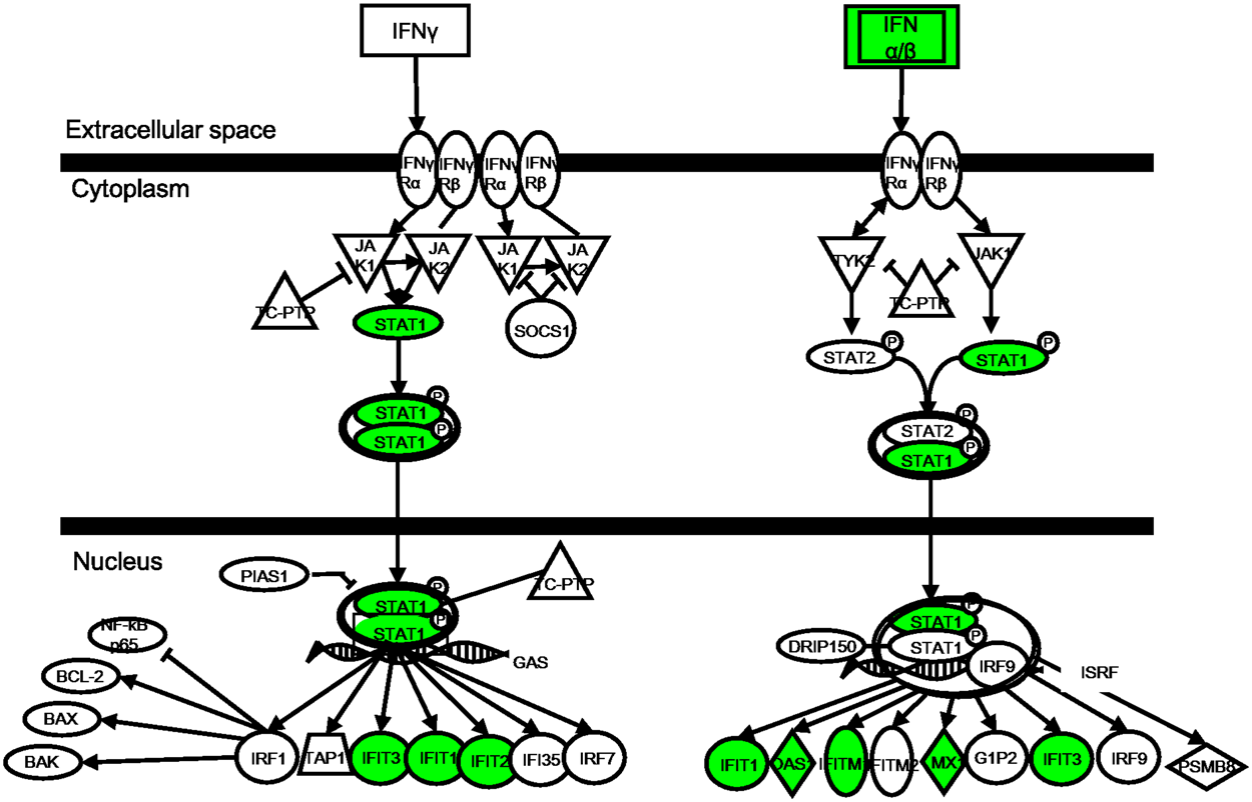
KLHDC7B affects genes involved in the “Interferon signaling pathway”. The 188 genes deregulated with an expression change higher than 1.5-fold were analyzed for the canonical pathway analysis by IPA. The highest confidence pathway is relevant to “Interferon Signaling Pathway”. Five (colored) of the 36 constituents in the pathway had significantly changed expression in the same direction to suppress the pathway (*P* = 2.9 × 10^−6^).

**Figure 7:**
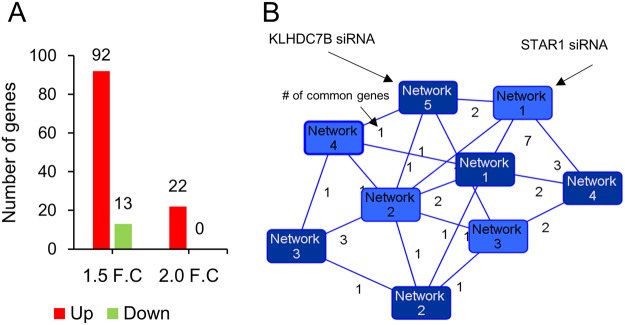
Network association between KLHDC7B and STAR1. (**A**) Number of genes statistically deregulated by STAR1 downregulation in microarray analysis. Number of genes showing higher than 1.5 (1.5 F.C) and 2.0 fold (2.0 F.C) is shown after up- and downregulation of STAR1. (**B**) IPA networks were constructed for deregulated genes by downregulation of KLHDC7B and STAR1. The top five networks were then compared to monitor common genes appearing in both networks. The dark blue and light blue-colored boxes denote for the network from KLHDC7B and STAR1, respectively. The number on the solid line indicates the number of genes that appear in common.

Many genes in the KLHDC7B-downregulated dataset are related, such as STAT1, IFIT3, and MX1, with the antimicrobial or infectious disease properties showing decreased expression. In detail, the top canonical pathway with the highest Z-score was the “Interferon signaling pathway”. Among the 36 genes in the pathway, eight were significantly changed with all their expression decreased to inhibit the pathway (Fig. 6). The “Interferon signaling pathway” was also revealed in the STAR1-deregulated cell by a few approaches. First, two of the seven genes in the network association study, Oligoadenylate synthase-like (OASL) proteins and IFI27, which hit both the top network of KLHDC7B-downregulation and STAR1-downregulation were expressed with an opposite dysregulation (Figs 1 and 7, Supplementary Figs S1B and S4). These are known to be involved in a defense system to protect against viruses^20^ or to be stimulated by interferon^21^. Second, ten interferon signaling-related genes in Fig. 1A obtained from the KLHDC7B-downregulation were examined by RT-PCR for RNA from the STAR1-upregulated MCF-7. As a result, expression of six genes was in the same direction of the KLHDC7B-downregulation (Supplementary Fig. S5), supporting the theory that KLHDC7B and STAR1 function through opposing mechanisms on the cancer cell activity through interferon signaling.

## Discussion

In the current study, we aimed to clarify the roles of KLHDC7B and STAR1 lncRNA in the molecular events during the proliferation of breast cancer cells. The molecular and cellular impact of dysregulation of KLHDC7B was remarkably stronger when the gene was downregulated rather than upregulated. This was demonstrated by the larger number of genes affected and more severely reduced proliferation by downregulation of KLHDC7B. Other tumor-related genes showed a similar result. For example, 296 genes were affected when FLRT2, which had tumor suppressor activity in breast cancer was downregulated by siRNA; only 92 genes were affected when an overexpression was introduced^22^. A threshold for protein expression level of the gene may exist; therefore, an overdose of the protein does not accompany a prominent change. Meanwhile, a shortage of the protein causes a severe impact on protein expression.

So far, a few action modes of lncRNA are known, including cis-acting and trans-acting ones^23^. An example of cis-acting lncRNA is lincRNA-p21 that influences the p53 tumor suppressor pathway by acting in cis as a locus-restricted coactivator for p53-mediated p21 expression^24^. In order to examine the possible cis-acting activity of STAR1, expression of genes nearby STAR1 was screened from the microarray data. The ST8SIA8, LOC105376436, and LOC105376437 were found around STAR1, however, no gene showed a significant expression change, suggesting a low possibility of cis-acting mode (data not shown). For the trans-acting mode, lncRNA can act after combining with its partner protein. In case of lncRNA HOTAIR, the RNA interacts with Polycomb Repressive Complex 2 (PRC2), affecting breast cancer progression and survival through regulation of a variety of genes such as HOXD, the progesterone receptor, and SNAIL^25^. When STAR1 was examined *in silico* for its binding potential to proteins using the catRAPID program (http://s.tartaglialab.com/page/catrapid_group), a few candidate proteins such as NT5C, BEX5, and RBM18 emerged with a high probability. However, our preliminary pull-down experiments using a biotin-labeled STAR1 RNA failed to identify any interaction between STAR1 and a specific protein. A further systematic approach is needed to reveal whether STAR1 acts alone or with other RNA-binding proteins.

It appears uncommon for two genes, KLHDC7B and STAR1, to show opposite effects on the cell proliferation or apoptosis while their expression is significantly associated in breast cancer. The two genes may carry out different roles during carcinogenesis, i.e. KLHDC7B may control cell proliferation while STAR1 may control cancer cell migration. This scenario is supported by a few observations in this study. First, cell proliferation effect was prominent only when KLHDC7B was dysregulated, especially by siRNA rather than by the over-expression vector. However, overexpression or downregulation of STAR1 in cancer cells has a negligible effect on proliferation. Second, the alteration of cell migration was observed when STAR1 rather than KLHDC7B was dysregulated. Third, the top network represented by the affected genes after STAR1 was inhibited by siRNA included a few cancer cell migration-related genes such as STAT3^26^, MAP3K8^27^, and GDF15^28^. All these genes may stimulate metastasis in various cancers, and notably, their expression was opposite or not significantly changed in the KLHDC7B-downregulated cells.

In the cascade of the signaling pathway, STAR1 lies downstream of KLHDC7B because the STAR1 expression fluctuated according to the deregulated KLHDC7B, but not for KLHDC7B when STAR1 was dysregulated. Further study needs to discover how KLHDC7B is activated and induces STAR1. The STAT1-mediated interferon signaling pathway appears central to the KLHDC7B signaling pathway. This fact was supported by results showing STAT1 and many of its interferon-mediated target genes including IFIT1, IFIT2, IFIT3, OAS1, IFITM1, and MX1 were downregulated when KLHDC7B was induced to be downregulated (Fig. 6). STAT1 has been known to contribute to tumor development in various cancer types through complicated ways. A preponderance of *in vivo* data suggests that STAT1 acts as a tumor suppressor in preventing breast tumor initiation^29^. Paradoxically, when expressed at very high levels, STAT1 may also promote metastasis and drug-resistance^30^. The IFIT1, IFIT2, and IFIT3 are components of an IFN-induced protein with tetratricopeptide repeats (IFIT) family members. IFITs preferentially bind to mis- or un-modified viral RNA in the cytoplasm to block its efficient translation, playing a key role in anti-viral activity^31^. Expression of IFITs is downregulated in hepatocellular carcinoma^32^. The OAS1 gene is a proapoptotic as well as an antiproliferative gene that converts adenosine triphosphate into a series of 2′–5′ oligoadenylates^33^. MX1 is a key mediator of the interferon-induced antiviral response and is downregulated in cancer cells. Ectopic MX1 expression decreases motility, invasiveness, and metastasis of cancer cells, suggesting the protein as a tumor suppressor^34^. The opposite expression pattern of all these genes in the STAR1-downregulated cell may contribute to the explanation of the different effects of STAR1 and KLHDC7B during carcinogenesis.

In conclusion, a lncRNA, STAR1, was upregulated in breast cancer and its expression showed a significant association with KLHDC7B; dysregulation accompanied the alteration of STAR1 expression. The two genes are involved in breast cancer development, with KLHDC7B acting on proliferation and STAR1 on cell migration. They could be used as molecular markers for breast cancer diagnosis. In addition, through identifying the casts involved in their signaling pathways, they could contribute to the complete understanding of the molecular activities of the two genes during carcinogenesis.

## Materials and Methods

### Cell culture and transfection

Four human mammary gland-derived cell lines (MCF-10A, MCF-12A, MDA-MB-231, and MCF-7) were purchased from the American Type Culture Collection (ATCC, Manassas, VA, USA). MCF-10A and MCF-12A were cultured in MEGM (Lonza, Basel, Switzerland) with 100 ng/ml cholera toxin. MDA-MB-231 and MCF-7 were cultured in RPMI1640 (Gibco, Grand Island, NY, USA) supplemented with 10% FBS and 1% penicillin and streptomycin. A KLHDC7B-expressing plasmid vector harboring full-length cDNA in pReceiver-M02 was purchased from Genecopoeia (Rockville, MD, USA; Cat. No.: EX-T2688-M02). For STAR1-expressing vector, a DNA fragment spanning the cDNA (GenBank No.: NR_034129.1) was chemically synthesized (Genecopoeia) and subcloned into the pcDNA 3.1/Hygro(+) (Invitrogen, Carlsbad, CA, USA). The siRNAs of KLHDC7B and STAR1 were designed by Bioneer (Daejeon, South Korea) and Qiagen (Redwood City, CA, USA), respectively. Plasmid vectors and siRNAs were transfected with Lipofectamine 3000 and lipofectamin RNAIMAX (Invitrogen) following the supplier instructions.

### Study subjects

Breast cancer tissues used in this study are the same as ones adopted in our previous study^35^. All patients provided written informed consent to donate removed tissue to the National Cancer Center (NCC) in Korea. Samples were obtained according to protocols approved by the Research Ethics Board of the NCC.

### Real-time RT-PCR

Total RNA from cultured cells in 75-cm^2^ and approximately 200 mg of tissues was isolated using the ZR-DuetDNA/RNA MiniPrep (Zymo Research, Irvine, CA, USA) with a final elution in 45 μl of distilled water. Reverse transcription was conducted using 2 μg of total RNA with a ReverTra Ace qPCR RT MasterMix with gDNA Remover kit (Toyobo, Osaka, Japan). Gene expression was measured using a Kapa SYBR Fast qPCR Kit (Kapa Biosystems, Wilmington, MA, USA) on an ABI 7300 instrument (Applied Biosystems, Foster City, CA, USA). Each of the samples was performed in triplicate. GAPDH was used for normalization of each gene expression. The 2^−ΔCt^ method was used for the quantifying process, where ΔCt = (Ct_TARGET_ − Ct_GAPDH_). Primer sequences used for PCR are shown in the Supplementary Table S1.

### Genome-wide expression analysis

An Illumina HumanHT-12 v4 Expression BeadChip (Illumina, San Diego, CA, USA) analysis containing 47,231 probes was processed by Macrogen (Seoul, Korea). Briefly, biotinylated cDNA was prepared from total RNA using the Illumina RNA amplification kit (Ambion, Austin, TX, USA). Fluorescent signals were obtained by an Illumina BeadArray Reader, and data extraction and analysis were performed with an Illumina BeadStudio Application. Data were processed by excluding genes with *P*-values over 0.05, and differentially expressed genes were obtained displaying at least a 1.5-fold difference between the control cells and the tested cells. The array data were uploaded to the Gene Expression Omnibus (GEO) database (http://www.ncbi.nlm.nih.gov/geo/) with accession numbers GSE112518 and GSE112517 for overexpression and knock-down data of KLHDC7B and GSE112519 for knock-down data of STAR1. Significantly changed gene pools obtained from expression array were further analyzed with Ingenuity Pathway Analysis (IPA) (Qiagen; www.qiagen.com/ingenuity) to construct disease and function annotation, and network map. Each disease and function were listed based on the hierarchical order of the activation Z-score. The top network was nominated with the highest confidence among the networks.

### Cell proliferation assays

A cell proliferation assay was carried out using a CCK-8 kit (Dojindo, Kumamoto, Japan) following the manufacturer’s instructions. Briefly, 3 × 10^4^ cells/mL were seeded onto each well of an optically clear 96-well plate and cultured up to eight days. Colorimetric absorbance was measured at 450 nm with a microplate reader 2 h after incubation with 10 μl of CCK-8 solution on the day of examination.

### Flow cytometric analysis

For detection of proliferation or apoptosis, cells were transfected with an expression vector or a siRNA and incubated for 48 h. For analyzing proliferation, cells were fixed in ice-cold ethanol overnight. Ribonuclease A was then added to a final concentration of 100 μg/ml to degrade the RNA of fixed cells. Propidium iodide (PI) was added to a final concentration of 50 μg/ml and incubated in a darkened room for 5 min before analyzing with flow cytometry. To analyze apoptotic cells, FITC Annexin V Apoptosis Detection Kit I (BD Biosciences, Franklin Lakes, NJ, USA) was used following the manufacturer’s instructions. Prepared cells were analyzed on an Accuri C6 (BD Biosciences) and the data were evaluated with Accuri C6 Software (BD Biosciences).

### Migration assay

The migration assay was performed using a 6.5 mm Transwell apparatus with 8.0 µm pore polycarbonate membrane chamber system (Corning Inc., Corning, NY). There were 0.5~1.0 × 10^5^ starved cells seeded in the upper chamber in a 0.1 ml serum-free medium; a 0.6 ml medium with 10% fetal bovine serum was placed at the lower well, which was used as a chemoattractant. After incubation for 24 h at 37 °C, the migrated cells on the lower surface of the chamber were stained with 0.01% crystal violate and counted using Image J software.

### Statistical analysis

A Mann-Whitney U-test was used to evaluate the expression difference between normal and cancer tissue samples. The R-value was obtained by using Spearman’s correlation to calculate the correlation between KLHDC7B and STAR1 expression in breast tissue samples. All experimental results were performed at least three times independently and analyzed by the two-sided Student’s t-test. A *P*-value < 0.05 was considered statistically significant.

## Electronic supplementary material

Supplementary Information
