# Supplementary material for: A Kelch domain-containing KLHDC7B and a long non-coding RNA ST8SIA6-AS1 act oppositely on breast cancer cell proliferation via the interferon signaling pathway
Source: Sci Rep. 2018 Aug 27;8:12922. doi: 10.1038/s41598-018-31306-8 (PMC6110865; doi:10.1038/s41598-018-31306-8)

A Kelch domain-containing KLHDC7B and a long non-coding RNA ST8SIA6-AS1 act oppositely on breast cancer cell proliferation via the interferon signaling pathway

Gookjoo Jeong, Hansol Bae, Dawoon Jeong, Juyeon Ham, Sungbin Park, Hyeon Woo Kim, Han-Sung Kang, and Sun Jung Kim

Supporting Information

Table S1. Information of primers employed in this study

Gene Symbol	Sequence (5´- 3´)	Supplier
GAPDH	F : ACATCGCTCAGACACCATG<br>R : TGTAGTTGAGGTCAATGAAGGG	IDT
KLHDC7B	F : CCTGGGCAACACCATTTACT<br>R : GGGCAGAGTCAGGATGAATG	Genotech
STAR1	F : ATCGGCATGCCTAATACGTC<br>R : AAATGTGTGCTGGCAATTCTT	
IFIT1	F : GAATAGCCAGATCTCAGAGGAG<br>R : TGATCATCACCATTTGTACTCA	
IFIT2	F : GCAACCATGAGTGAGAACAATAAG<br>R : TCCATCAAGTTCCAGGTGAAAT	
IFIT3	F : TCATGAGTGAGGTCACCAAGAAT<br>R : CACACTCTATCTTCTAGATCCCTTGAG	
IL29	F : AGGCCTGTATCCAGCCTCAG<br>R : AGCCAGCGGACTCCTTTT	
IRF7	F : GGGCAAGTGCAAGGTGTA<br>R : GAAGACTCTGAAGTCGAAGATGG	
ISG15	F : GAGAGGCAGCGAACTCATCT<br>R : CTTCAGCTCTGACACCGACA	
MX1	F : CCCATATTTCAGGGATCTGC<br>R : CAGGGGCAGAGATTTACAGA	
OAS2	F : CGAGGAGAAGCTGTGTATCTATTG<br>R : ATTACTGGCCTCGCTGATTG	
STAT1	F : ACAAGGTGGCAGGATGTCTC<br>R : TGCTCCCAGTCTTGCTTTTC	
STAT2	F : GGACTTTGGTTACCTGACTCTG<br>R : GATGATGTGCAGTTCCTCTGT	

Figure S1. Induction of deregulation of KLHDC7B and STAR1 in cell lines. Cultured cells were transiently transfected with a siRNA to induce downregulation or an ORF to induce upregulation of the indicated gene. (**A**) Dysregulation of KLHDC7B in MCF-7. (**B**) Dysregulation of STAR1 in MCF-7. (**C**) Dysregulation of KLHDC7B in MDA-MB-231. (**D**) Dysregulation of STAR1 in MDA-MB-231. Expression of the gene was examined by real-time RT-PCR. GAPDH was used for normalization of each gene expression. The $2^{-\Delta Ct}$ method was used for the quantifying process. The result is depicted as Mean ± SE. NC, negative control ORF vector, or siRNA; ORF, open reading frame-containing cDNA; KLH, KLHDC7B.

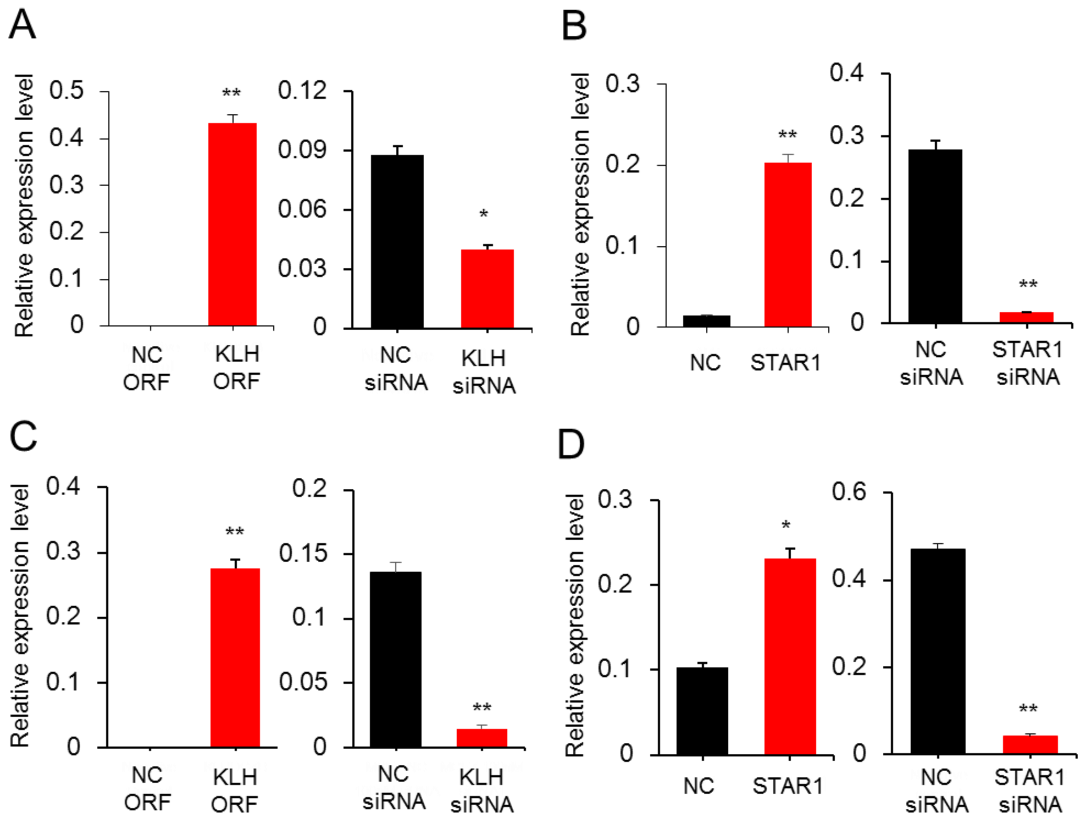

Figure S2. STAR1 is upregulated by KLHDC7B and shows upregulation in breast cancer lines. (**A**) STAR1 is one of the three genes, RBM3, LOC100128098 (STAR1), and ZNF254, that commonly appeared in the microarray analysis after induction of downregulation and upregulation of KLHDC7B in MCF-7. The number on the bars in the diagram shows the number of genes upregulated (**red**) or downregulated (**green**) by a siRNA (**blue diagram**) or an ORF (**red diagram**) of KLHDC7B. (**B**) Expression of STAR1 was examined in normal (**black bar**) and cancer cell lines (**red bar**) and depicted as Mean ± SE of three independent experiments.

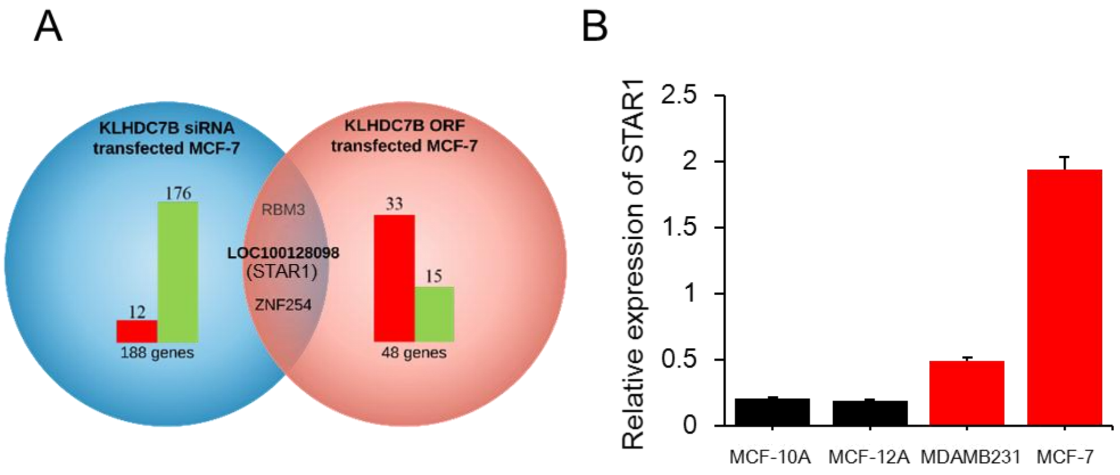

Figure S3. Effect of KLHDC7B and STAR1 on cell cycle of MCF-7. KLHDC7B and STAR1 were upregulated (**A** and **C**) or downregulated (**B** and **D**) by an ORF or a siRNA, and the cells were analyzed by FACS. NC, negative control vector, or siRNA.

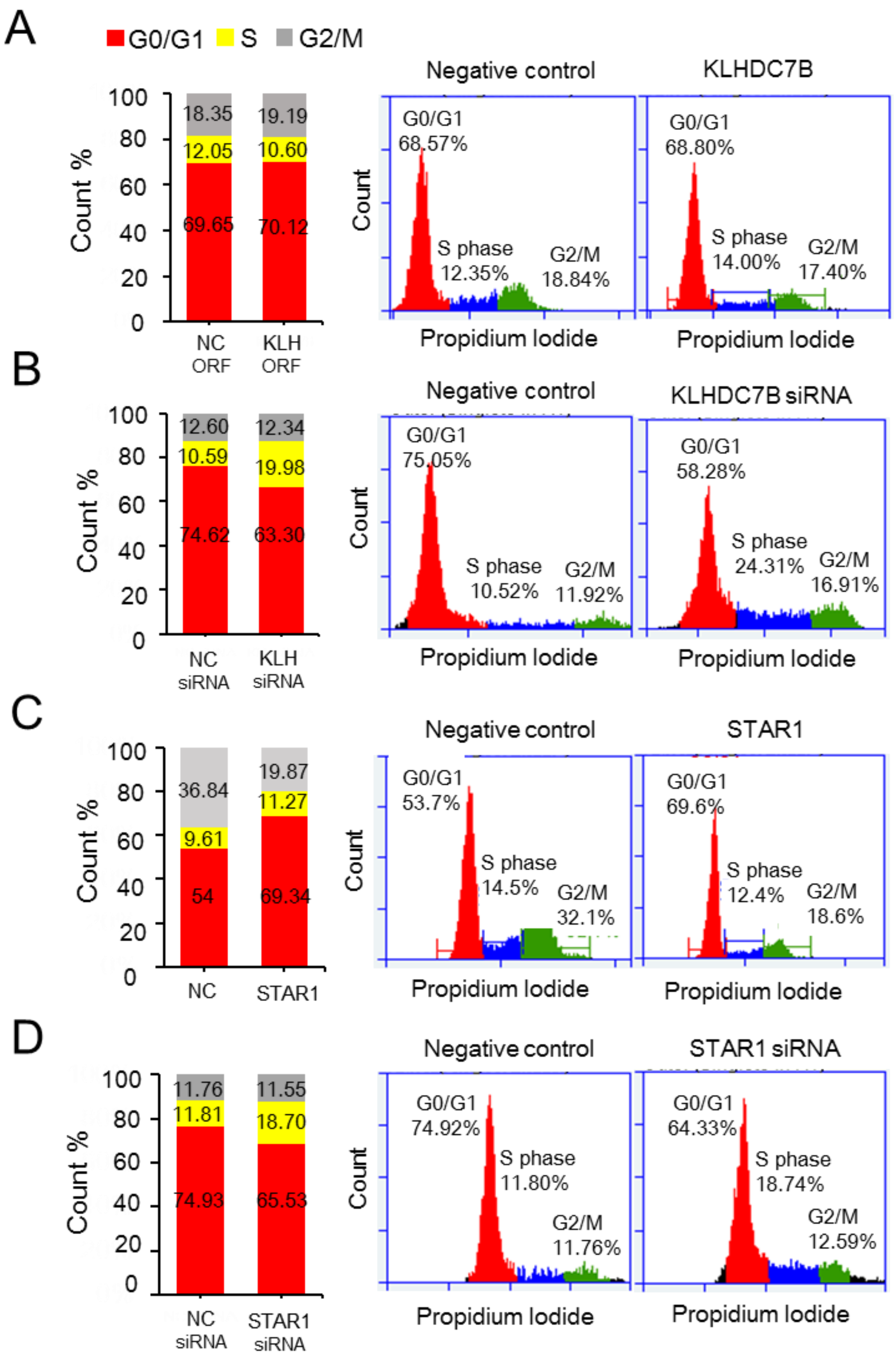

Figure S4. Highest confidence network of genes displaying altered expression by downregulation of STAR1 in MCF-7. STAR1 was downregulated in MCF-7 by transiently transfecting a siRNA and microarray analysis was performed. The 99 genes that were deregulated with an expression change higher than 1.5-fold were analyzed by IPA. The highest confidence network is relevant to "Cardiovascular Disease, Cellular Development, Cellular Growth, and Proliferation". The meaning of shapes and color and lines are as indicated in the legend of **Fig. 1**.

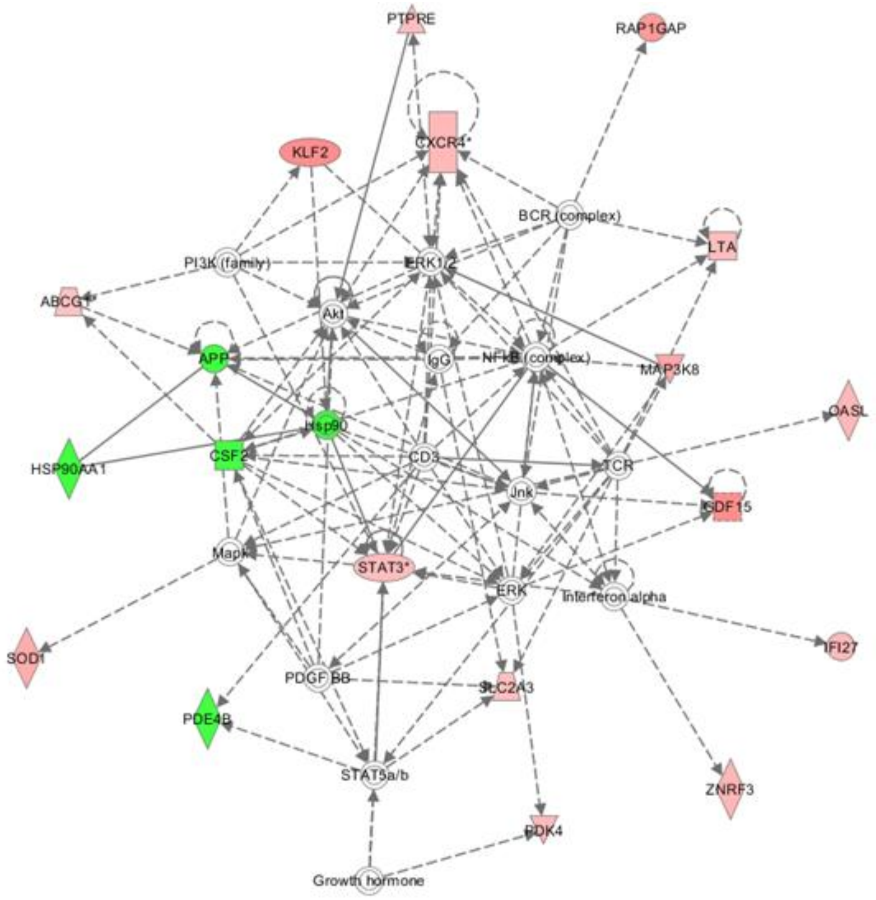

Figure S5. RT-PCR analysis of genes in the interferon signaling pathway being regulated by KLHDC7B and STAR1. Expression of the ten genes in the interferon signaling pathway, which has shown deregulation by KLHDC7B and STAR1, was examined by RT-PCR and depicted as Mean ± SE of three independent experiments. **Black bar**, RNA of control vector-transfected MCF-7; **red bar**, RNA of STAR1-containing vector transfected MCF-7.

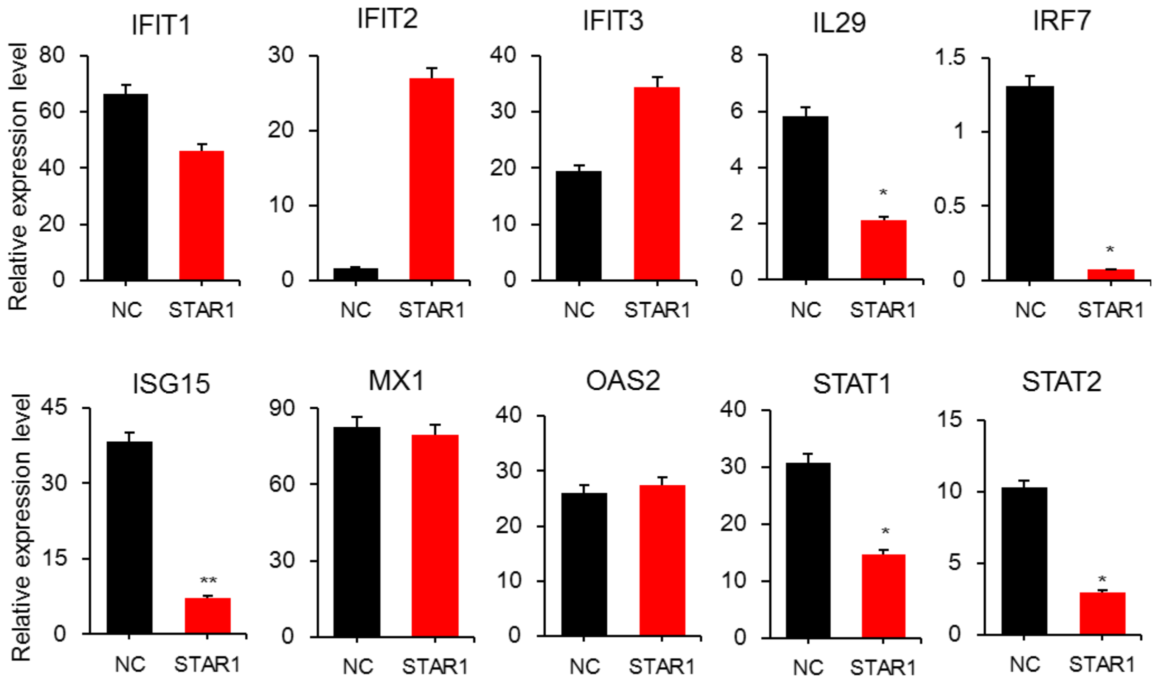

Supplement: Supplementary file 1 — Supplementary Information [file 41598_2018_31306_MOESM1_ESM.pdf]
